# Signaling Mechanisms and Disrupted Cytoskeleton in the Diphenyl Ditelluride Neurotoxicity

**DOI:** 10.1155/2014/458601

**Published:** 2014-06-22

**Authors:** Regina Pessoa-Pureur, Luana Heimfarth, João B. Rocha

**Affiliations:** ^1^Departamento de Bioquímica, Instituto de Ciências Básicas da Saúde, UFRGS, 90035-003 Porto Alegre, RS, Brazil; ^2^Departamento de Bioquímica e Biologia Molecular, Centro de Ciências Naturais e Exatas, Universidade Federal de Santa Maria, 97105-900 Santa Maria, RS, Brazil

## Abstract

Evidence from our group supports that diphenyl ditelluride (PhTe)_2_ neurotoxicity depends on modulation of signaling pathways initiated at the plasma membrane. The (PhTe)_2_-evoked signal is transduced downstream of voltage-dependent Ca^2+^ channels (VDCC), N-methyl-D-aspartate receptors (NMDA), or metabotropic glutamate receptors activation via different kinase pathways (protein kinase A, phospholipase C/protein kinase C, mitogen-activated protein kinases (MAPKs), and Akt signaling pathway). Among the most relevant cues of misregulated signaling mechanisms evoked by (PhTe)_2_ is the cytoskeleton of neural cells. The *in vivo* and *in vitro* exposure to (PhTe)_2_ induce hyperphosphorylation/hypophosphorylation of neuronal and glial intermediate filament (IF) proteins (neurofilaments and glial fibrillary acidic protein, resp.) in different brain structures of young rats. Phosphorylation of IFs at specific sites modulates their association/disassociation and interferes with important physiological roles, such as axonal transport. Disrupted cytoskeleton is a crucial marker of neurodegeneration and is associated with reactive astrogliosis and apoptotic cell death. This review focuses the current knowledge and important results on the mechanisms of (PhTe)_2_ neurotoxicity with special emphasis on the cytoskeletal proteins and their differential regulation by kinases/phosphatases and Ca^2+^-mediated mechanisms in developmental rat brain. We propose that the disrupted cytoskeletal homeostasis could support brain damage provoked by this neurotoxicant.

## 1. Introduction

Tellurium (Te) is an element sharing the same group of sulfur and selenium in the periodic table; that is, it is the heaviest of the stable chalcogens (group 16) and is classified as a metalloid. In contrast to oxygen, sulfur, and selenium, tellurium has no essential physiological role in cell biology [1]. However, due to its chemical versatility, Te has been extensively used in chemistry, particularly, in organic synthesis (for comprehensive reviews about the importance of tellurium in organic synthesis, see [2–6]). In addition to its use in organic synthesis, tellurium is widely used in the vulcanization of rubber and in metal-oxidizing solutions to tarnish metals, such as silver. More recently, tellurium (as CdTe, HgTe, and PbTe) has also been used in the composition of quantum dots (QD) in thermoelectric materials, in digital versatile disk-random access memory (DVD-RAM), and in DVD-recordable disks (DVD-RW) [7–9]. The presence of tellurium in different types of electronic materials and nanomaterials is an important health issue. These materials usually contain a variety of toxic elements and there is a paucity of research about the environmental and occupational toxicity of those materials [10–13]. Most importantly, the fate of electronic material constituents is unknown, but tellurium can be released in the environment either as elemental tellurium or as more reactive cation forms. The toxicity of elemental tellurium and its ionic forms have also been little explored in the literature [14, 15]. After its release in the environment, tellurium can be biomethylated to more volatile intermediates and, consequently, can be mobilized from soil or from aquatic bodies to the atmosphere [10, 16]. In short, the presence of tellurium in the environment is expected to increase in the next years or decades.

**Figure 1:**
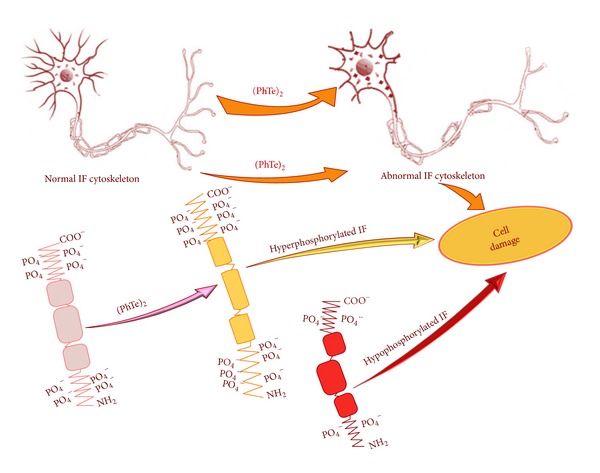
Schematic representation of disrupted intermediate filament (IF) phosphorylation. The hyper- or hypophosphorylation of IFs can change the architecture of the cytoskeleton and lead to cell damage.

Here in this review, we will give emphasis to diphenyl ditelluride, the simplest of the diaryl ditellurides, which is used as an intermediate in organic synthesis [17]. This organic compound of tellurium, diphenyl ditelluride or (PhTe)_2_, has been described to possess very contrasting and interesting biological activities [18–23], including antioxidant properties stronger than its selenium analog, the diphenyl diselenide or (PhSe)_2_ [23]. However, the toxicological properties of this compound seem to be more striking than its potential pharmacological properties (for review, see [17]). Despite this, we must emphasize that the question of tellurium toxicity may be related to the stability of the carbon–tellurium bond (C–Te bond) [24–29]. For instance, we have observed that diethyl 2-phenyl-2 tellurophenyl vinylphosphonate (DPTVP) was nontoxic to mice when tested at doses much higher than that of (PhTe)_2_ [26], which indicated that perhaps some organic chemical forms of tellurium can be safe for therapeutic use. Indeed, there are some indications in the literature that tellurium could be of potential pharmacological importance (see, for instance, [30–39]). Unfortunately, the rational study of tellurium toxicity is incipient and there is no systematic study of organoselenium and organotellurium toxicity. The approach to this important question has been limited to few laboratories and most of the studies are empirical in nature [17, 29, 37, 38, 40–46]. Thus, the progress in the field of organochalcogen compounds as potential pharmacological agents will require new more refined approaches other than simple empirical testing of new compounds (see, for instance, [45]). In order to offer elements to support rational protocols to study the toxicity and pharmacology of organochalcogens, particularly tellurides, we have been investigating the* in vitro* and* in vivo* neurotoxicity of the simplest and the prototypal of the diaryl ditelluride molecules, that is, (PhTe)_2_ in rats, using the intermediate filaments as the targets of organotellurium toxicity (Figure 1).

Most importantly, evidence from our group in the last years has shown that, beyond the gross neurotoxic actions described in the CNS of rodents [17],* in vivo* exposure to (PhTe)_2_ and* ex vivo* quantification of telluride effects on neural cells of rats indicated serious disruption of the homeostasis of cytoskeletal proteins in a spatiotemporal manner. Neurofilaments (NFs), microtubule-associated proteins (MAPs), and Tau make up the dynamic cytoskeletal architecture of neurons. Phosphorylation/dephosphorylation of these cytoskeletal proteins is a dynamic process regulated by many kinases/phosphatases which, in turn, are downstream of multiple signaling cascades. Aberrantly phosphorylated cytoskeletal proteins are associated with disrupted cytoskeleton, characteristic of neuronal damage in several human neurodegenerative diseases. Consequently, in this review, we will focus on recent insights into the neurotoxic actions of (PhTe)_2_ on the cytoskeleton. Particularly, we will consider experimental evidence of the signal transduction pathways involved in the misregulation of the phosphorylating system associated with intermediate filament (IF) proteins in neural cells. We will focus on studies performed* in vivo* and in semi-intact brain preparations, such as acute brain slices in view of the preservation of an integrated communication network.

## 2. An Overview of Tellurium Toxicity

The toxicity of tellurium has been little explored in the literature. Indeed, the exposure of humans to tellurium has been rare [1, 14, 17] and this perhaps explains the paucity in the studies of tellurium toxicity. However, as indicated above, the use of tellurium in organic synthesis, the debate that organic forms of tellurium could have pharmacological and therapeutic properties [30–34], and its use in electronic materials indicate that more studies about the molecular mechanisms of its toxicity are needed, particularly, in view of its potential neurotoxicity in mammals [1, 17, 43, 47–49].

**Figure 2:**
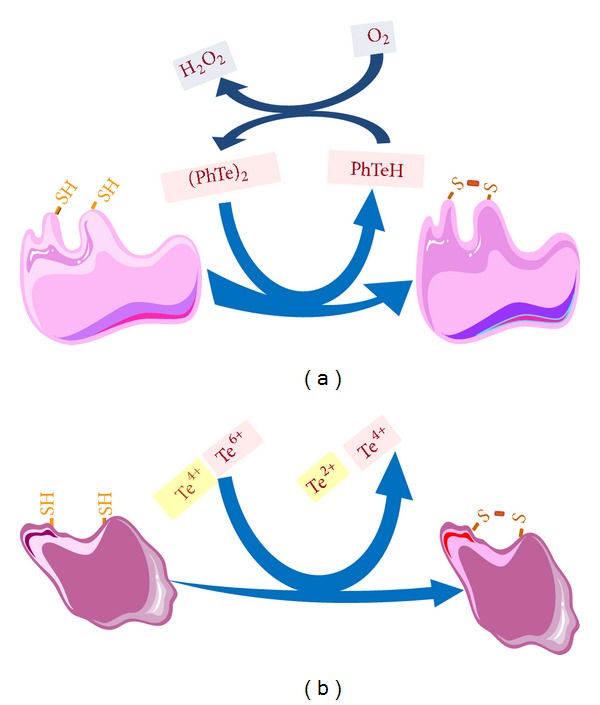
Oxidation of thiol groups from targets proteins by diphenyl ditelluride [(PhTe)_2_] (a) or by cationic forms of tellurium (Te^4+^ and Te^6+^) (b). The oxidation of thiol groups can be catalytic, because the reduced form of ditelluride (tellurol) can be easily oxidized back to (PhTe)_2_ by molecular oxygen.

**Figure 3:**
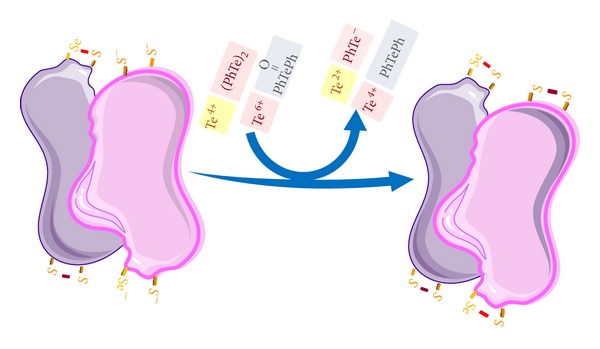
Hypothetical oxidation of thiol and selenol groups from thioredoxin reductase (TrxR) dimers by inorganic (Te^4+^ and Te^6+^) and organic (diphenyl ditelluride [(PhTe)_2_] and ditelluroxide). Different tellurium forms can target both the two vicinal thiol or thiol-selenol groups in different points of the functional asymmetric dimer of the enzyme.

The identification of tellurium molecular and cellular targets is highly desirable from toxicological, physiological, and biochemical point of view. In the case of mammals, the target organ or system seems to vary depending on the type and the developmental stage of tellurium exposure. Reports of acute human intoxication are rare and there are only 2 cases of heavy intoxication of adults (see below) and in those cases, the administration of a solution containing the inorganic Te(IV) targeted multiple tissues [50]. However, at that time, no molecular endpoint of toxicity was assessed [50]. Tellurium in its elemental form can be either reduced to Te^−2^ or oxidized to Te^+4^ or Te^+6^ [51]. The anionic reduced form of tellurium is very unstable and the cationic forms are much more stable than that of Te^−2^. Indeed, Te(IV) and Te(VI) can interact with thiol groups and this interaction is involved in the toxicity of inorganic tellurite and tellurate (Figure 2). Tellurium in organic molecules can also undergo redox chemistry after interacting and oxidizing thiol groups. Thus, in view of its big size, tellurium behaves more as redox active metal than a nonmetal. This explains the ability of its cationic forms to oxidize thiolates and possibly selenolates [52–57]. In a similar way to selenite (Se(IV)), tellurite can inhibit thiol containing enzymes and this can be the molecular basis of tellurium toxicity (Figure 2). In the case of elemental tellurium, inhibition of squalene monooxidase, which has thiol groups in its active site, is an important step in tellurium neurotoxicity [17]. Since elemental tellurium is expected to have “weak chemical reactivity,” its toxic effects after interacting with living cells must require its oxidation to cationic forms (Te(IV) and Te(VI)). Recently, we have demonstrated that some important selenoenzymes (for instance, glutathione peroxidase and thioredoxin reductase) can be targeted after exposure to diphenyl ditelluride [57] (Figure 3). Indeed, some organic forms of tellurium have been indicated as potential anticancer agents via inhibition of the selenoenzyme thioredoxin reductase [58–61]. The literature has also indicated that tellurium can replace selenium in some selenium proteins or amino acids [62, 63]. However, the extent or the existence of such substitution after* in vivo* exposure to tellurium has not been explored in detail.

## 3. Neurotoxicity of Inorganic Tellurium in Rodents

Elemental tellurium ingestion is toxic to rats and short-term exposure to high levels of Te^0^ has been associated with transitory segmental demyelination of different types of fibers [65] and the inhibition of cholesterol synthesis, at the level of squalene epoxidase, in Schwann cells is an important metabolic process in Te-induced demyelination [49, 66]. One key and primary ultrastructural observation in tellurium induced neuropathy is the presence of cytoplasmic lipid droplets in myelinating Schwann cells [48]. The chemical form of tellurium involved in squalene monooxygenase inhibition is unknown but tellurite (Te(IV), dimethyltellurium dichloride, and dimethyltelluride can inhibit the brain enzyme from rats [47, 67]. The mechanism of squalene monooxygenase enzyme inhibition by methyltelluride and tellurite is related to the oxidation of vicinal thiols in the enzyme (Figure 2). Other studies have also indicated the oxidation of endogenous thiol groups by organotellurium compounds [68, 69]. In effect, in a similar way to inorganic tellurium compounds, thiol-containing proteins seem to be preferential targets of organotellurium intoxication [17, 54–56, 69–74] (Figure 2(b)).

Developing brain is particularly sensitive to exogenous chemical agents [75–77]. Accordingly, the gestational exposure of rats to tellurium is associated with hydrocephalus [78–81], but not in rabbits [81]. However, the relation of hydrocephalus with cholesterol synthesis inhibition is not well defined. It is interesting to note that exposure of adult mice to (PhTe)_2_ was associated with the appearance of spherical, spongeous-like holes in the brain [17, 82]. In contrast to inorganic tellurium exposure [83], they were not characterized by the presence of lipofuscin [82].

One prototypal and interesting tellurium compound that has been studied in the literature is the inorganic tellurium molecule called AS-101 [34–39, 84]. This compound has interesting immunomodulatory properties and it was even proposed to be used in the treatment of AIDS [82] via inhibition of reverse transcriptase and immunomodulating properties [34]. The topical application of AS-101 has been demonstrated to stimulate the growth of hair and its use in the treatment of baldness was suggested in the past [39]. Despite these interesting observations, the therapeutic use of AS-101 is still a promise. The reasons for this are certainly related to the toxicity of this compound. In effect, the intoxication with AS-101 caused a myriad of signs of intoxication in rats, including reduction in food consumption and body weight, the presence of hind limb paresis, hematological changes, and histopathological alteration in several organs [85]. It is interesting to point out that we have observed that exposure of mice to high doses of (PhTe)_2_ was associated with hind limb paresis (unpublished observations). Furthermore, the typical garlic odor was reported after exposure to the inorganic AS-101 [85] and organic (PhTe)_2_. In short, the toxicity of tellurium in either the inorganic or organic form has been little explored. At the molecular level, the redox transition from elemental or anionic tellurium to Te(IV), followed by interactions with endogenous thiol groups of low molecular weight molecules or with critical proteins, can explain the toxicity of tellurium. However, we have limited knowledge about the preferential protein targets of tellurium. Indeed, proteins having vicinal groups such as squalene monoepoxide and ALA-D [17, 84] can be important targets of tellurium intoxication. But the identification of primary target(s) of tellurium is still elusive. Here, we will show that (PhTe)_2_ disrupts the signaling pathways associated with phosphorylation and dephosphorylation of cytoskeletal elements in the central nervous system of rats, which can also be related to modulation of the redox state of some critical proteins by the tellurium atoms found in (PhTe)_2_.

## 4. Cases of Human Exposure to Tellurium

In two adults exposed accidentally to high quantities of sodium tellurite, the most characteristic observations were the presence of a garlic odor in the breath and cyanose, which were associated with methaemoglobin in the blood. These patients were exposed to about 2 g of sodium tellurite after retrograde pyelography examination, where sodium iodide was inadvertently replaced by sodium telluride. The presence of alkyltelluride (methyltelluride) was reported in one of the two patients that were lethally intoxicated with tellurite. Furthermore, the blood and/or the organs of the patients also exhaled a garlic odor. The color of organs was modified by the intoxication, reflecting possibly the deposition of elemental tellurium. Histological examination of different tissues demonstrated fatty degeneration and edema, which was more marked in liver than in brain, lungs, kidneys, spleen, and heart [86]. A third patient, who survived the minor intoxication with the same solution that killed the two other patients, received only small amount of sodium tellurite, because, on injecting the supposed “sodium iodide” solution, the catheter was blocked and withdrawn. He developed the typical garlic odor, but no additional symptoms of tellurium intoxication.

It is interesting to point out that acute or subacute exposure of mice to high doses of (PhTe)_2_ caused changes in the color of different organs and also the presence of garlic odor exhales from the body and organs of heavily intoxicated mice (unpublished observations). The garlic odor indicates that (PhTe)_2_ can release Te atom, which is metabolized to methylated forms of Te.

Less severe and nonfatal exposure to tellurium was reported in 2 children who ingested metal-oxidizing solutions containing tellurium. These solutions are normally used to clean silver objects [87] and were accidentally ingested by the children. Clinical signals of intoxication included vomiting, black discoloration of the oral mucosa, and garlic odor to the breath, which in one of the young children persisted for several months after the intoxication. The presence of garlic odor, though not a definitive clinical signal of tellurium exposure, should be considered as an important clinical feature by the health agents and may assist clinicians in the diagnosis of rare tellurium poisoning [87]. Two postgraduates investigating the potential therapeutic or industrial use of tellurium esters were exposed to tellurium hexafluoride gas in the laboratory [88]. The symptoms of intoxication included tellurium metallic taste in the mouth and presence of the typical garlic odor garlic in the breath, sweat, and urine. One of them became anorexic. Furthermore, they exhibited bluish-black patches in the webs of the fingers and, to a lesser degree, in the skin [88].

From a clinical point of view, it is important to emphasize that exposure of humans to levels of tellurium that did not produce overt signs of toxicity, for instance, after occupational exposure, was associated with garlic odor [89]. Furthermore, urinary levels of tellurium higher than 1 *μ*mol/mol creatine were associated with a higher likelihood of garlic odour reporting [89]. Thus, garlic odor, though not a conclusive cue, can help the physician in identifying intoxication or nonclinical exposure to tellurium.

## 5. An Overview on the Neurotoxicity of Diphenyl Ditelluride

Diphenyl ditelluride is the simplest of the aromatic, diorganoyl ditelluride compounds. It has been used in organic synthesis for a long time and in the last 15 years its toxicity and, particularly, neurotoxicity have been extensively studied. Indeed, developmental exposure to (PhTe)_2_ is teratogenic [17, 19, 90] and has been associated with long-term behavioral and neurochemical changes in rats [20–22, 91]. However, the majority of those studies have investigated the phenomena involved in the toxicity of (PhTe)_2_, but the molecular mechanism(s) involved in (PhTe)_2_ effects has (have) not been studied in detail. The general toxicity of (PhTe)_2_ seems to be related to oxidation of thiol-containing proteins as depicted in Figure 2(a) (for a comprehensive review, see [17, 69]). In this review, we will present the recent findings from our laboratory indicating that signaling mechanisms involved in regulating intermediate filament phosphorylation/dephosphorylation are important targets of (PhTe)_2_. Our results also indicate that (PhTe)_2_ intoxication can be used to mimic some molecular changes found in important brain pathologies. Consequently, (PhTe)_2_ can be used as a tool to study pharmacological agents that could counteract the toxic effect of ditelluride on brain phosphorylating/dephosphorylating system, for instance, organoselenium compounds with neuroprotective effects [17, 92, 93].

## 6. Physiology and Pathophysiology of the Cytoskeleton

The cytoskeleton, consisting of microtubules, intermediate filaments (IFs), and actin filaments, is indispensable for any eukaryotic cell. Cytoskeleton networks form complex intracellular structures that vary during the cell cycle and between different cell types according to their physiological role. IF proteins constitute the third main cytoskeletal system of vertebrate cells, expressed in a tissue- and development-specific manner.

According to the degree of sequence identity, IF proteins have been grouped into six sequence homology classes (SHC): acidic keratins (SHC I); basic keratins (SHC II); desmin; vimentin; and other mesenchymal IF proteins such as glial fibrillary acidic protein (GFAP) (SHC III); neurofilament (NF) proteins (SHC IV); lamins (SHC V); and an orphan group harboring the lens specific IF proteins phakinin and filensin [94, 95]. All of the IF proteins are considered to provide structural and mechanical support to the cell and are also involved in multiple cellular functions, including transport, protein and organelle targeting, migration, signaling, apoptosis, and protection from stress [96].

Neurofilaments are the neuron specific IFs. They consist of three subunits divided according to their molecular mass: NF heavy chain (NF-H: 190 kDa), NF middle chain (NF-M: 115 kDa), and NF light chain (NF-L: 68 kDa). In common with other members of the IF family, NF-H, NF-M, and NF-L each comprise a central alpha helical coil-coiled rod domain flanked by a variant amino-terminal globular head domain and a hypervariable carboxyl-terminal tail domain which differ in length among the subunits [97–99]. The tail domains of NF-M and NF-H are longer than the other IF proteins and extensively phosphorylated, forming lateral projections along the NF axis, responsible for NF spacing [100–102]. Thus, one of the functions of NFs in neurons is to control the axonal caliber and consequently nerve conductivity since the speed of conductivity of a nerve impulse is directly proportional to the caliber of the axon [103]. Also, myelination determines conduction velocity in larger axons and this is too associated with an increase in side-arm phosphorylation in NF-M and NF-H [104–108].

Neurofilaments are transported from the cell body where they are synthesized, to be delivered along the axon by a mechanism called axonal transport [109, 110]. The motors implicated in the anterograde transport are known to be kinesins, while the retrograde transport is mediated in association with dyneins, the same motor proteins involved in the fast axonal transport along microtubules [111]. It is known that carboxyl-terminal phosphorylation of NF-H progressively restricts association of NFs with kinesin and stimulates its interaction with dynein [112]. This event could represent one of the mechanisms by which aberrant carboxyl-terminal phosphorylation would slow NF axonal transport.

Astrocytes are important cytoarchitectural elements of the CNS; however, during the past few years, molecular and functional characterization of astroglial cells indicate that they have a much broader function than only supporting the neurons in the brain, as they have specialized functions in inducing and regulating the blood brain barrier (BBB), glutamate uptake, synaptic transmission, plasticity [113], and metabolic homeostasis of the brain [114, 115]. Glial fibrillary acidic protein (GFAP) is the main IF protein expressed in mature astrocytes, where it is thought to help maintaining mechanical strength as well as the shape of cells. However, recent evidence has shown that GFAP plays a role in a variety of additional astrocyte functions, such as cell motility/migration, cell proliferation, glutamate homeostasis, neurite outgrowth, and injury/protection [113].

Because of their multiple roles in the cells, cytoskeletal protein components are among the main targets modified in response to extracellular signals that ultimately determine cell morphology and physiological role [116]. Consequently, it is not surprising that IFs are likely to be targeted in several genetically determined protein misfolding/aggregation diseases [117–119] as well as by a variety of pathogens [120] and toxins [92, 93].

Abnormally accumulated NFs are a pathological hallmark of many human neurodegenerative disorders, including amyotrophic lateral sclerosis [121], Alzheimer's disease [122, 123], Parkinson's disease [124, 125], Charcot-Marie-Tooth [126], giant axonal neuropathy [127], neuronal intermediate filament inclusion disease [128, 129], and diabetic neuropathy [130, 131]. Multiple factors can potentially induce the accumulation of NF, including dysregulation of NF gene expression, NF mutations, defective axonal transport, abnormal posttranslational modifications, and proteolysis [132]. Beyond their association with neural damage in inherited or age-dependent neurodegenerative diseases, the disruption of NF homeostasis has been reported in response to toxic agents, such as beta, beta′-iminodipropionitrile (IDPN) [133–135], aluminium chloride [136], and methylmercury [92].

Astrocytes are also involved in a wide range of CNS pathologies, including trauma, ischaemia, and neurodegeneration. In such situations, the cells change both their morphology and expression of many genes leading to activation of astroglia, or astrogliosis [113, 137, 138]. Astrogliosis is characterized by the increase of IFs with accompanying cellular hypertrophy and an abnormal apparent increase in the number of astrocytes. Upregulation of IF proteins, in particular GFAP, but also vimentin and nestin, two IF proteins that are abundantly expressed in immature astrocytes, is regarded as the hallmark of astrogliosis [137, 139]. However, the most remarkable evidence of the relevance of GFAP in the physiological roles of astrocytes in maintaining normal brain function is Alexander disease, the devastating leukodystrophy resulting from dominantly acting mutations in the coding region of GFAP [140]. These mutations have been associated with the presence of Rosenthal fibers, referring to intracellular protein aggregates containing GFAP and stress proteins in astrocytes [141].

## 7. Roles of Phosphorylation in the Intermediate Filament Homeostasis

IF proteins are known to be phosphorylated on their head and tail domains and the dynamics of their phosphorylation/dephosphorylation plays a major role in regulating the structural organization and function of IFs in a cell- and tissue-specific manner [142–146]. Amino-terminal phosphorylation plays a major role in regulating the assembly/disassembly equilibrium of type III IFs as well as of NF-L and NF-M subunits of NFs [147].* In vivo* and* ex vivo* studies from our group and others demonstrated that the phosphate groups on the amino-terminal head domain on GFAP, vimentin, and NF-L are added by second messenger-dependent protein kinases, such as cAMP-dependent protein kinase (PKA), Ca^2+^/calmodulin-dependent protein kinase II (PKCaMII), and protein kinase C(PKC) [147–152]. Phosphorylation of Ser-8, Thr-7, Ser-13, and Ser-38 in the N-terminal region (head domain) of GFAP [153–155] causes disassembly of the IFs and, conversely, the action of protein phosphatases leading to dephosphorylation restores its ability to polymerize [155]. Moreover, in the C-terminal region (tail domain), phosphorylation of Ser-389 affects the interactions between GFAP and other IF proteins [156]. GFAP phosphorylation is possibly a key factor in astrocytes, since cells use phosphorylation/dephosphorylation levels to regulate the dynamic of the polymerization/depolymerization of these proteins promoting cell survival and physiological roles.

The assembly of NFs into a heteropolymer is dependent on the head domains of NF-L and NF-M and more specifically on the phosphorylation level of these domains. The major sites of* in vivo *phosphorylation on NF-L and NF-M subunits were identified to be Ser-55 (PKA) [149] and Ser-23 (PKA, PKC) [157, 158].* In vitro* results point to Ser-57 (PKCaMII) [149, 159], Ser-12, Ser-27, Ser-33, and Ser-51 (PKC) [160] as the main amino-terminal phosphorylation sites on NF subunits.

NFs are synthesized in the cell body, but they are extensively phosphorylated after they are transported into the axon [109, 110]. Phosphorylation sites in the tail domain of NF-M and NF-H are found in a glutamic acid rich region with Ser residues. These Ser residues can be phosphorylated by casein kinases I and II on the basis of their consensus sequences [161, 162]. However, NF-M and NF-H are extensively phosphorylated in phosphorylation sites located in Lys-Ser-Pro (KSP) repeat regions of the tail domain of these subunits. Phosphorylation of the KSP repeats rends NF-H the most extensively phosphorylated molecule in the nervous system [163]. The KSP repeats are phosphorylated by proline-directed kinases such as Cdk5 [164], the mitogen-activated protein kinases (MAPK) such as Erk1/2, JNK, and p38MAPK [165–168], and glycogen synthase kinase 3 (GSK3) [167–169].

The phosphorylation of NFs occurs in close proximity to myelin sheaths [168]. Thus, myelination may be the signal needed to induce phosphorylation of NFs in axons and it is also possible that a signal from Schwann cells or oligodendrocytes might be related with Cdk5 and MAPK activation. The carboxyl-terminal tail regions of NF-M and NF-H protrude laterally from the filament backbone to form “side-arms” when phosphorylated. Phosphorylation of these sites regulates the interactions of NFs with each other and with other cytoskeletal structures, mediating the formation of a cytoskeletal lattice that supports the mature axon [143, 145].

Moreover, carboxyl-terminal phosphorylation of NF-M and NF-H subunits has long been considered to regulate their axonal transport rate and in doing so to provide stability to mature axons [147]. The NFs are transported in the slow axonal transport component [170, 171], which results from binding to the fast motor proteins kinesin and dynein intermitted with prolonged pauses [172–174].

Evidence accumulated from studies of our group in the last years point to a critical role of the endogenous phosphorylation of IF proteins in response to a variety of signals in both physiological and pathological conditions [116, 148, 149, 152, 175–177], highlighting the cytoskeleton as a preferential target of the signal transduction pathways. Importantly, a large body of experimental evidence shows a link between misregulation of cell signaling mechanisms, disruption of IF phosphorylation, and cell damage in response to different stress signals. While the exact signaling pathways regulating NF phosphorylation remain elusive, there is increasing evidence that known signal transduction cascades are involved. These actions can be initiated by the activation of N-methyl-D-aspartate- (NMDA-), voltage-dependent Ca^2+^ channels type L (L-VDCC), or G protein-coupled receptors, and the signal is transduced downstream of Ca^2+^ mobilization or monomeric GTPase activation through different kinase/phosphatase pathways, regulating the dynamics of the cytoskeleton [148, 149, 152, 176, 177].

## 8. Assessing the Molecular Basis of Diphenyl Ditelluride Toxicity on the Cytoskeleton of Neural Cells

The brain is one of the major targets of (PhTe)_2_ toxic actions [17] (see above in Section 5). To assess the effects of (PhTe)_2_ on the cytoskeleton of neural cells and to shed light onto the signaling cascades targeted by the neurotoxicant, we used pharmacological and immunological approaches in which specific enzyme inhibitors, channel blockers, or glutamate antagonists as well as monoclonal antibodies directed to signaling cascades or specific phosphorylation sites let us conclude that (PhTe)_2_ interferes with the cell signal transduction misregulating the phosphorylation/dephosphorylation of IFs and disrupting the homeostasis of the cytoskeleton of astrocytes and neurons. Compelling evidence points to a remarkable role of Ca^2+^ mediating these actions secondary to NMDA and L-VDCC activation.* In vivo *studies have demonstrated that disruption of the cytoskeleton takes part in the deleterious effects of (PhTe)_2_ on neural cells culminating with astrogliosis and neuronal death [177, 178]. Also, acute brain slices were useful to further elucidate the molecular basis of the (PhTe)_2_ neurotoxicity. In this context,* in vivo* and* ex vivo* studies have been important tools to shed light into the molecular mechanisms of the neurotoxicant on the cytoskeleton of neural cells [64, 178–183]. More details about the cascade of molecular events triggered by (PhTe)_2_ will be presented below.

## 9. Central Roles of Ca^2+^ and Glutamate Receptors Mediating the Actions of Diphenyl Ditelluride on the Cytoskeleton

Most of the actions of (PhTe)_2_ disrupting the homeostasis of the cytoskeleton in neural cells are mediated by high Ca^2+^ levels. Changes in the cytoplasmic free Ca^2+^ concentration constitute one of the main pathways by which information is transferred from extracellular signals received by animal cells to intracellular sites [184–186]. Otherwise, an augmented Ca^2+^ influx through the NMDA receptor or VDCC can be responsible for the activation of lethal metabolic pathways. Different toxins and stress conditions [187–190] have been implicated in the regulation of intracellular Ca^2+^-dependent processes in cells, and different cell types exhibit a diverse range of transient responses to the same stimulus.

Exposure of tissue slices to (PhTe)_2_ triggers the activation of ionotropic and metabotropic glutamate receptors as well as VDCC and the endoplasmic reticulum Ca^2+^ channels. These receptors and channels activate several cellular responses by distinct signaling pathways in a spatiotemporally regulated manner. Metabolism of cyclic nucleotides and membrane phospholipids and activation of different protein kinases and phosphatases, particularly MAPKs, PKC, CaMKII, and protein phosphatase 1 (PP1) as well as endogenous enzymatic regulators, are the key biochemical steps and pathways we have evidenced. Alterations in these key brain mechanisms disrupt the homeostasis of the cytoskeleton and this could explain the neural damage observed following* in vivo* exposure to (PhTe)_2_ [179].

Intracellular Ca^2+^ is one of the crucial signals that modulate the action of (PhTe)_2_ in rat brain. This is in line with previous evidence pointing dysregulation of Ca^2+^ homeostasis as an important event in driving different neuropathologies, such as in the expression of the malignant phenotypes [191] and in neurodegenerative conditions [192]. Interestingly, (PhTe)_2_ provoked different responses in the cerebral cortex and hippocampus [182], reinforcing that different types of cells can respond in a different way to the same extracellular signal molecule. Taking into account the compelling evidence of its ability in disrupting signaling mechanisms, ditelluride can be used as a tool to induce molecular changes similar to those found in different pathologies of the brain. Consequently, the study of its neurotoxicity can be instrumental to understand not only the basis of tellurium toxicity but also the role of pathways involved in the neuropathology of different types of brain diseases associated with aging.

**Figure 4:**
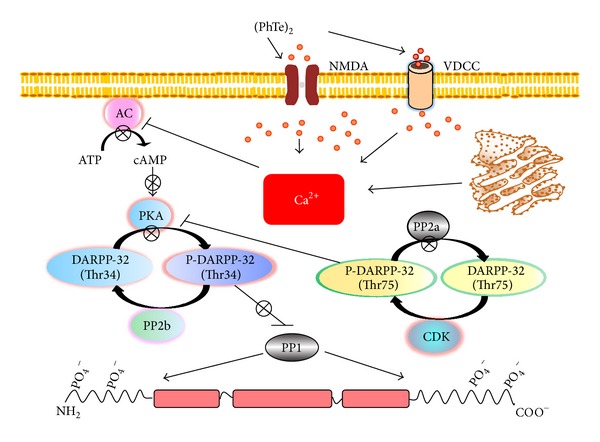
Schematic representation of the proposed mechanism of action of (PhTe)_2_ on the IF-associated phosphorylating system of cerebral cortex neural cells of young rats* in vitro*. (PhTe)_2_ acts in NMDA and VDCC channels, increasing intracellular Ca^+2^ levels. The second messenger directly inhibits AC and PKA activities, decreases the phosphorylation level of DARPP-32 (Thr-34), and releases PP1 activity. Taken together, these actions change the phosphorylation status of IF proteins* in vitro*. NMDA, N-methyl-D-aspartate receptor; VDCC, voltage-dependent calcium channel; PKA, cAMP-dependent protein kinase; DARPP-32, dopamine- and cAMP-regulated phosphoprotein, Mr 32 kDa; PP1, phosphoprotein phosphatase 1; PP2b, calcineurin; AC, adenylate cyclase. Orange-red circles represent Ca^+2^.

Acute brain slices exposed to (PhTe)_2_ showed PP1-mediated hypophosphorylation of GFAP and NF subunits in cerebral cortex of 9- and 15-day-old rats but not in hippocampus at this developmental period [182]. Hypophosphorylation was dependent on ionotropic glutamate receptors, L-VDCC, and ryanodine channels. Interestingly, activation of PP1 was modulated by dopamine and cyclic AMP-regulated neuronal phosphoprotein 32 (DARPP-32), an important endogenous Ca^2+^-mediated inhibitor of PP1 activity. Depending on the site of phosphorylation, DARPP-32 is able to produce opposing biochemical effects, that is, inhibition of PP1 activity or inhibition of protein kinase A (PKA) activity. Phosphorylation of DARPP-32 at Thr34 by PKA constitutes an important mechanism to activate DARPP-32, blocking PP1. Conversely, when pThr34 DARPP-32 is dephosphorylated by protein phosphatase 2B (PP2B), it is itself inhibited, promoting the release of PP1 activity. Moreover, phosphorylation of Thr75DARPP-32 by Cdk-5 inhibits PKA, reinforcing the release of PP1 activity [193] (Figure 4).** (**PhTe)_2_ induced decreased phosphorylation level of DARPP-32 at Thr34 and increased phosphorylation levels of DARPP-32 at Thr75. These findings are compatible with inactivation of PKA, releasing PP1 to dephosphorylate the IFs. It is interesting to note that the complexity of the neurotoxic effect evoked by (PhTe)_2_ in Cdk5 is involved in NF hypophosphorylation, rather than hyperphosphorylation, as previously described [194]. Decreased cAMP and PKA catalytic subunit support that (PhTe)_2_ disrupted the cytoskeletal associated phosphorylating/dephosphorylating system of neurons and astrocytes through PKA-mediated inactivation of DARPP-32, promoting PP1 release and hypophosphorylation of IF proteins of those neural cells (Figure 4). Buffering the intracellular Ca^2+^ by the Ca^2+^ chelator Bapta-AM showed that it is upstream of cAMP and PKA modulation [182]. During artificial or agonist-induced Ca^2+^ oscillations, Willoughby and Cooper [195] detected fast, periodic changes in type 8 adenylyl cyclase (AC8) with subsequent PKA-mediated phosphodiesterase 4 (PDE4) activity in human embryonic kidney (HEK293) cells. As corollary, it can be concluded that the dynamic complexity of Ca^2+^ signaling includes the ability of Ca^2+^ to regulate other second messengers, such as cAMP. In this context, our results corroborate the role of Ca^2+^ as an upstream effector of (PhTe)_2_-evoked signal on the cytoskeleton, since hypophosphorylation was abolished in the presence of NMDA antagonists (MK-801 and DL-AP5), L-VDCC (verapamil and nifedipine), and endoplasmic reticulum (dantrolene) Ca^2+^ channels [182] (Figure 4).

IF hypophosphorylation is in line with previous evidence showing that protein phosphatases are highly concentrated in the mammalian brain [196–198] and pointing the cytoskeleton as a preferential target of the action of phosphatases in both physiological and pathological conditions [199–201]. Moreover, hypophosphorylation of IF proteins could also be associated with brain damage, since the increased NF packing density correlates with decreased phosphorylation of KSP repeats in the carboxyl-terminal domains of NF-M and NF-H [202].

It is evident that the neurotoxic mechanism of (PhTe)_2_ in the cerebral cortex involves the state of phosphorylation of DARPP-32, a modulator of the cAMP pathway previously described to be highly expressed in striatal projection neurons [203]. It is interesting to note that we have previously described (PhTe)_2_-induced hyperphosphorylation in the cerebral cortex of 17-day-old rats* ex vivo *rather than hypophosphorylation, as observed in the hippocampus [93]. These paradoxical divergent findings provide an interesting insight into the differential susceptibility of cortical IF cytoskeleton to the exposure to this neurotoxicant and could reflect the existence of different vulnerabilities of the cytoskeleton of cortical cells during development based on the temporal maturation mediated by a multitude of developmental processes and signaling pathways [75, 204]. However, the exact explanation for the differential effects of (PhTe)_2_ on the cytoskeletal protein phosphorylation as a function of postnatal age remains to be clarified. Probably, they are associated with the pathological role of the developmentally regulated glutamate receptors in neural cells within the brain which is dependent on the maturation patterns of glutamate receptor expression [205, 206]. Although the developmentally regulated ontogenetic expression of the glutamate receptors is poorly known, it has been shown that the expression patterns of glutamate receptor subunit genes change during the ontogeny of the brain [207–209]. Distinct regional and temporal patterns of expression of types and subtypes of the glutamate ionotropic receptors during ontogeny [205, 206] may possibly explain the different signaling pathways targeting the cytoskeleton of cortical neural cells during development.

**Figure 5:**
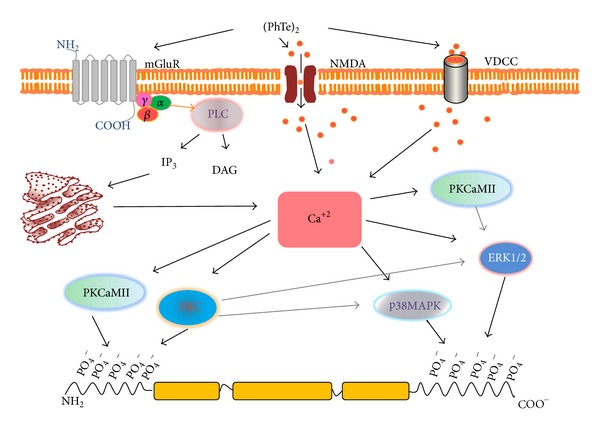
Schematic representation of the proposed mechanism of action of (PhTe)_2_ on the IF-associated phosphorylating system of hippocampus neural cells of young rats* in vitro*. (PhTe)_2_ acts through mGluR, NMDA, and VDCC channel, increasing intracellular Ca^+2^ levels. The second messenger directly activates PKCaMII and PKC and indirectly activates ERK1/2 and p38MAPK. Taken together, these actions change the phosphorylation status of IF proteins* in vitro*. mGluR, glutamatergic metabotropic receptor; NMDA, N-methyl-D-aspartate receptor; VDCC, voltage-dependent calcium channel; PKC, protein kinase C; PKA, ERK, extracellular-signal-regulated kinase, PKaMII, Ca^+2^/calmodulin-dependent protein kinase II. Orange-red circles represent Ca^+2^.

In contrast with cerebral cortex,* ex vivo *exposure to (PhTe)_2_ (100 *μ*M) induced hyperphosphorylation of astrocyte and neuron IFs in acute hippocampal slices of 21-day-old rats. Hyperphosphorylation is partially dependent on L-VDCC, NMDA, and endoplasmic reticulum Ca^2+^ channels. The role of Ca^2+^ as an upstream signal of this effect was demonstrated by specific NMDA antagonists and Ca^2+^ channel blockers as well as extra- and intracellular Ca^2+^ buffering, which totally prevented IF hyperphosphorylation [181] (Figure 5).

The signal evoked by (PhTe)_2_ is also transduced through metabotropic glutamate receptors on the plasma membrane leading to the activation of phospholipase C (PLC) which catalyses the hydrolysis of phosphatidylinositol 4,5-bisphosphate (PIP2) to produce the intracellular messengers inositol 1,4,5-trisphosphate (IP3) and diacylglycerol (DAG). IP3 is highly mobile in the cytoplasm and diffuses into the cell interior where it binds to specific receptors on the endoplasmic reticulum. The binding of IP3 changes the conformation of IP3 receptors such that an integral channel is opened. Ca^2+^ stored at high concentrations in the endoplasmic reticulum is released to the cytoplasm. Therefore, high Ca^2+^ levels and DAG directly activate PKCaMII and PCK, resulting in the hyperphosphorylation of Ser-57 in the carboxyl-terminal tail domain of NF-L. Also, the activation of Erk1/2 and p38MAPK resulted in hyperphosphorylation of KSP repeats of the medium molecular weight NF subunit (NF-M). It is noteworthy that PKCaMII and PKC inhibitors (KN-93 and staurosporine, resp.) prevented (PhTe)_2_-induced Erk1/2MAPK and p38MAPK activation as well as hyperphosphorylation of KSP repeats on NF-M, suggesting that PKCaMII and PKC could be upstream of this activation [181]. Interestingly, this effect implies a significant cross-talk among signaling pathways elicited by (PhTe)_2_, connecting the glutamate metabotropic cascade with activation of Ca^2+^ channels. The final molecular result is the extensive phosphorylation of amino- and carboxyl-terminal sites on IF proteins [181]. This is in line with the proposal that hyperactivation/deregulation of these kinase cascades may induce aberrant phosphorylation of the cytoskeletal proteins in neurons leading to neural dysfunctions seen in neurodegenerative diseases [210].

## 10. Diphenyl Ditelluride Disrupts the Cytoskeleton and Provokes Neurodegeneration in Acutely Injected Young Rats

The brain has a prolonged period of postnatal maturation, and myelination is not complete until adolescence [211], which in the rat brain is up to postnatal day 50 [212]. The initial appearance and progressive phosphorylation of NF-M and NF-H in the axons are region-specific and appear to be correlated to synaptogenesis and myelination, as the mature axonal cytoskeleton begins to be established [213, 214]. Therefore, it is expected that the deleterious effects of tellurium are preferentially expressed during development, since the intense plasticity underlying the developmental events [215, 216] is dependent on efficient remodeling of the cytoskeleton which, in turn, is dependent on the physiological phosphorylation of the cytoskeletal proteins. Improper developmental plasticity likely impedes the normal information processing in the brain. In line with this,* in vivo* exposure to (PhTe)_2_, in which the neurotoxicant reaches the brain via systemic circulation, provokes aberrant phosphorylation of IF proteins from neural cells by MAPK (Erk, JNK, and p38MAPK) and PKA activities, as demonstrated in the striatum [179] and cerebellum of young rats [64].

According to the* ex vivo* evidence, the phosphorylating system associated with the cytoskeleton from different brain regions of developing rats also shows different susceptibilities to* in vivo* (PhTe)_2_ exposure. This can be evidenced in cerebral cortex and hippocampus of 15-day-old rats acutely injected with a toxic dose of (PhTe)_2_ (0.3 *μ*mol/kg body weight) [20, 180]. Cortical hyperphosphorylation of neuronal and glial IF proteins was an early and persistent event up to 6 days after injection, accompanied by increased levels of GFAP and NF-L. Upregulation of gene expression as well as GFAP and vimentin hyperphosphorylation could be a response to injury and take part in the program of reactive astrogliosis as demonstrated in striatum and cerebellum of (PhTe)_2_-injected rats [64, 179]. Otherwise, in the hippocampus the aberrant phosphorylation was a later response presented only by the astrocyte IFs, without detectable alteration of their levels into the cell, suggesting a milder response [180]. Taking into account the cytoskeletal response evoked by (PhTe)_2_, hippocampal astrocytes and neurons showed lower vulnerability than their cortical counterparts. These findings could be related to the differential pathophysiological responses of cortical and hippocampal neurons and astrocytes to the insult. Apparently, hippocampus of rodents is more resistant than cerebral cortex to other neurotoxic insults. This is supported by findings from [217] who described neuropathological changes and tau hyperphosphorylation in the cerebral cortex of adult mice exposed to methyl mercury, but not in the hippocampus.

Under a deleterious process, astrocytes become reactive releasing a wide array of mediators, including pro- and anti-inflammatory cytokines, neurotrophic factors chemokines, complement factors, and reactive oxygen species (ROS), all of which potentially mediate neuroprotective and/or neurotoxic effects [218–220]. In line with this, a strong evidence supports an important role of astrocytes in a more severe cortical than hippocampal damage following the* in vivo *(PhTe)_2_ insult.

The phosphorylation level of IF proteins from acute cortical slices from 18- and 21-day-old rats exposed to (PhTe)_2_ was not altered, while IFs of acute cortical slices from younger pups (9 and 15 days old) were hypophosphorylated. In addition, hippocampal IFs were not responsive to the insult until weaning [182]. In a sense,* ex vivo *and* in vivo *findings are in agreement, showing that cortical cells are more susceptible to the toxicant than their hippocampal counterparts. On the other hand, these results highlight the relevance of the interplay between glial and neuronal cells to adapt the cellular metabolic response to the insult. The loss of physiological brain connections in brain slices could underlie this response. These findings are in line with the role of astrocytes in determining the vulnerability of neurons to deleterious stimuli [175, 221, 222].

Aberrant phosphorylation of GFAP is associated with injury and pathological conditions. Similarly, abnormal phosphorylation of NFs is associated with neurodegeneration [148, 149, 223, 224]. In neurons, hyperphosphorylated NFs can inhibit their proteolytic breakdown by calpain, a Ca^2+^-activated protease [101, 225]. Abnormally phosphorylated NFs accumulate in the perikarya and the phospho-NF aggregates can thus become cytotoxic by the enduring impairment of axonal transport of NFs [226, 227]. In line with this, kinesin and dynein motor proteins were found to accumulate in NF spheroids in spinal motor neurons and spinal sensory ganglion neurons of chicks injected with beta′-iminodipropionitrile (IDPN) [228], which could impede the transport of components required for axonal maintenance, as demonstrated in a transgenic mouse model of amyotrophic lateral sclerosis [229]. The increased time the NF spent in the cell body is thought to result in further aberrant phosphorylation [230] and may prevent them from entering the axon, resulting in a deleterious feedback loop [147].

Consistent with a critical role of disrupted cytoskeleton in brain damage, MAPK and PKA activation as well as astrogliosis take part in the early responses to the insult with (PhTe)_2_ in rat cerebellum [179] and striatum [64]. However, the most striking difference between cerebellum and striatum response to (PhTe)_2_ is that in cerebellum astrogliosis preceded the apoptotic neuronal death, while in striatum astrogliosis was a later response concomitant with neuronal damage without net neuronal loss, emphasizing the higher vulnerability of the cerebellum to this neurotoxic effect. The different windows of susceptibility leading to activation of MAPK pathway targeting the cytoskeleton in the cerebellum compared with those in the striatum could once more underlie the differential response of these structures to the injury.

Neurodegeneration in (PhTe)_2_ rats was also related with inhibited Akt, activated caspase 3, and decreased [^3^H]glutamate uptake, emphasizing a critical role of altered Ca^2+^ levels in this process, since IP3 and ryanodine receptors may be important sensors of disturbed intracellular Ca^2+^ homeostasis [231]. On the basis of our findings, we propose that (PhTe)_2_ produced alterations in Ca^2+^ homeostasis and glutamatergic neurotransmission which lead to excitotoxicity and neurodegeneration in the developing rat brain.

**Figure 6:**
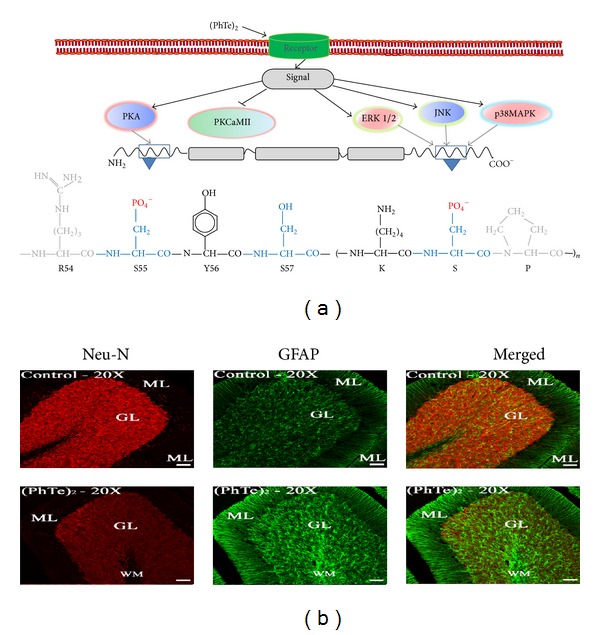
Effects of the* in vivo* treatment with (PhTe)_2_ on neural cells of young rats. (a) Schematic representation of the proposed mechanism of (PhTe)_2_-induced disruption of the IF-associated phosphorylating system in brain of young rats* in vivo*. (PhTe)_2_ activates cell surface receptors eliciting signaling cascades through intracellular second messengers, which activate the cyclic AMP- and Ca^2+^/calmodulin-dependent protein kinases (PKA and PKCaMII, resp.). Also, MAP kinases (Erk1/2; JNK, and p38MAPK) are activated, targeting specific sites on IF subunits. PKA and PKCaMII phosphorylate serine sites, such as Ser55 on NF-L, and MAPKs are directed to KSP repeats on the C-terminal domain on NF-M and NF-H. The hyperphosphorylated N-terminal domain misregulates the association/disassociation equilibrium of the filaments, while C-terminal hyperphosphorylation disrupts the interaction of the filaments with other cytoskeletal elements and with motor proteins. In box, chemical structure of phosphorylated amino acids. (b) The cerebellar damage induced by the* in vivo* exposure to (PhTe)_2_. Increased GFAP staining, one of the main features of reactive astrogliosis, is concomitant with decreased NeuN positive cells, indicative of reduced neuronal cells. Adapted from Heimfarth et al., 2013 [64].

Taking into account the great deal of evidence of NF hyperphosphorylation evoked in cerebral cortex, cerebellum, and striatum of (PhTe)_2_-injected rats, it is feasible that this neurotoxicant is not able to provoke demyelination in the CNS, in contrast with peripheral demyelination described in rats [20, 21, 232]. In this context, the specific molecules in myelinating cells that regulate signaling cascades, which in turn modulate the expression and phosphorylation of cytoskeleton elements, have not been fully identified. However, compelling evidence points to a role for myelin-associated proteins modulating expression and phosphorylation of NFs and consequently the axonal caliber [168, 202, 233]. A schematic representation of the* in vivo* actions of (PhTe)_2_ in the brain of young rats is depicted in Figure 6.

## 11. Gestational and Lactational Toxicity of Diphenyl Ditelluride

There are experimental points of evidence that (PhTe)_2_ causes gestational and lactational toxicity [19, 90]. Considering the lipophilic character of this compound, we can suppose that it could cross the placental barrier during pregnancy and be excreted in milk after birth, like other hydrophobic toxicants, such as polychlorinated biphenyls [234]. Thus, the characteristics of solubility of (PhTe)_2_ together with the high vulnerability during a period of intense brain development define the high vulnerability of perinatal rat brain as important targets of intoxication with Te.

The immature brain is a much more dynamically active tissue than the mature brain. Its high degree of plasticity and a broad range of potential developmental directions underlie developmental toxicity studies, indicating that maternal toxicity during the pregnancy and/or lactation has been shown to produce adverse effects in offspring [235]. Although the consequences of gestational and lactational exposure to any toxin on brain function are not well understood, exposure to neurotoxic chemicals is of particular concern when it occurs during early development. Fetal and neonatal brain development is characterized by developmental time windows during which certain brain regions or neuron types are specifically sensitive to environmental influences [236] and neurotoxicants [237].

Evidence that the toxic effects of maternal exposure to (PhTe)_2_ could be detected in their offspring has been previously reported. High doses of (PhTe)_2_ (0.12 mg/kg of body weight) can be teratogenic to rat and mice fetuses, causing malformations in fore- and hind-limbs, absent or short tail, subcutaneous blood clots, exophthalmia, hydrocephalus, and presence of exposed brain. However, the mice are less susceptible to toxic effects induced by (PhTe)_2_ than the rats, suggesting a different developmental toxicity induced by (PhTe)_2_ among species [19, 90].

Stangherlin et al. [22] demonstrated that subchronic exposure to (PhTe)_2_ (0.03 mg/kg of body weight), via maternal milk, caused oxidative stress in brain structures of young rats. The exposure to (PhTe)_2_ increased lipid peroxidation and inhibited *δ*-aminolevulinate dehydratase (*δ*-ALA-D), catalase, and superoxide dismutase (SOD) activities in hippocampus and striatum of young rats. Moreover, dam exposure to the same concentration of (PhTe)_2_ induced changes in the levels of nonenzymatic defenses in cerebral cortex and striatum of the lactating rats. Supporting the relevance of maternal milk as an important via of toxicity with the (PhTe)_2_, exposure of dams to low levels of (PhTe)_2_ during the first 14 days of lactation causes neurobehavioral changes of their offspring, which emphasizes the potential neurotoxicity of organic tellurium compounds [21]. Disrupted cytoskeleton could underlie these behavioral impairments since (PhTe)_2_ (0.01 mg/kg of body weight) evoked aberrantly phosphorylated astrocyte and neuron IFs observed on PND 15 and 21 in striatum and cerebellum of rats. On the other hand, exposure to (PhTe)_2_ via maternal milk provoked hyperphosphorylation of IF protein in the hippocampus only on PND 21 and 30 (unpublished results), evidencing a latter effect in this brain structure. This is in line with* ex vivo* and* in vivo* studies reporting disrupted phosphorylation level of hippocampal IFs in 21-day-old rats [178, 183]. These findings could be related with the differential pathophysiological responses of the different structures to the insult according to their critical proliferative period [212].

The effect of (PhTe)_2_ was spatiotemporally regulated, and the posttraductional mechanisms regulating the cytoskeleton from striatum and cerebellum in younger pups are more susceptible to the action of the neurotoxicant than in older ones. In fact, suckling rats can be considered extremely susceptible to (PhTe)_2_-induced neurotoxicity, since the dose of (PhTe)_2_ given to dams was extremely low. As a corollary, the offspring of (PhTe)_2_-treated dams is expected to be exposed to telluride levels much lower than those given to their mothers.

Further evidence of the great susceptibility of pups to the toxic effects of (PhTe)_2_ via maternal milk comes from the previously reported inhibition of glutamate uptake and Na^+^/K^+^ATPase activity in the brain of pups from exposed dams [22]. Moreover, MAPK and PKA pathways are activated in neural cells of striatum and cerebellum of the offspring [183]. This could be related to the disruption of the cytoskeleton observed in these pups. Interestingly, the kinase activities as well as the phosphorylating level of the IF proteins are increased in PND 15 and 21 and returned to control levels in older pups. This could be explained by adaptative mechanisms overriding the prolonged exposure to the toxicant; however, this point remains to be clarified. Also, we could presume that altered glutamate uptake and Na^+^/K^+^ATPase activity as well as disrupted cytoskeleton could be involved in the neurobehavioral changes of the offspring.

Taking into account the whole evidence that, following maternal exposure, (PhTe)_2_ reaches the brain of their offspring via systemic circulation, we are tempted to propose that results from the animal model could mimic the risk of a potential (PhTe)_2_ exposure of pregnant or lactating women to their babies. However, little information about human intoxication with (PhTe)_2_ during pregnancy and lactation is available in the literature and further efforts will be necessary to understand the pathology of this compound.

## 12. Concluding Remarks

The major questions concerning the (PhTe)_2_ toxicity that remain posed relate to the mechanisms that underlie their actions in the CNS. To address this question, we have focused our efforts on the actions of this neurotoxicant targeting the endogenous phosphorylating system associated with the cytoskeleton of neural cells of young rats. It is always expected that the deleterious effects of a toxin are preferentially expressed during development, since the intense plasticity underlying the developmental events [215, 216] is dependent on efficient remodeling of the cytoskeleton which, in turn, is dependent on the physiological phosphorylation of the cytoskeletal proteins. Improper developmental plasticity likely impedes information processing in the brain.

Compelling evidence from our group supports that IF proteins could be crucial in mediating spatiotemporal responses to (PhTe)_2_ in neural cells of rats. Central to its function is its dynamic phosphorylation and defects in the capacity to maintain the homeostasis of IF phosphorylation would probably have detrimental effects on cell function. These actions are complex, and integrated processes carefully and precisely orchestrated by the cell through membrane receptors and channels are able to activate diverse signal transduction pathways misregulating the homeostasis of the cytoskeleton. It is remarkable that Ca^2+^-mediated mechanisms were shown to play a central role in these membrane-initiated mechanisms. It is presumed that aberrantly phosphorylated/dephosphorylated IF proteins may interfere with neural cell structure and function which is associated with neurodegeneration in young animals. Further studies on membrane receptors and their downstream events might provide us with new insights into the molecular basis of the mechanisms triggered by the toxicant in brain. Although cell signaling transduction of (PhTe)_2_ does not have necessarily the same mechanisms in different brain regions, the Ca^2+^-initiated events highlight a role for this neurotoxicant as a disruptor of the cytoskeleton.

Taking into account the relevance of the signaling mechanisms targeting the cytoskeleton during early postnatal brain development, we presume that the spatiotemporal misregulation of the homeostasis of the cytoskeleton we evidenced can probably contribute to the deleterious action of (PhTe)_2_ on the developing brain, a fact that might explain at least in part the neurotoxicity of this compound; however, these consequences need further investigation. One aspect that needs further investigation is the effect of inorganic tellurium on IF phosphorylation. This is particularly relevant in view of the increased industrial use of tellurium in electronic devices.

We think that our studies in injected rats or in acute brain slices have made important contributions to our understanding of (PhTe)_2_-mediated injury. These studies provide the opportunity to examine cellular, molecular, and morphological changes after exposure to the neurotoxicant. However, extrapolation of conclusions from animal data to human beings must be done with caution. Of particular experimental importance, the study of (PhTe)_2_ neurotoxicity can be instrumental to understand not only the basis of tellurium toxicity but also the role of pathways involved in the neuropathology of different types of brain diseases associated with neurodegeneration and aging.
